# An integration of complementary strategies for gene-expression analysis to reveal novel therapeutic opportunities for breast cancer

**DOI:** 10.1186/bcr2344

**Published:** 2009-07-28

**Authors:** Andrea H Bild, Joel S Parker, Adam M Gustafson, Chaitanya R Acharya, Katherine A Hoadley, Carey Anders, P Kelly Marcom, Lisa A Carey, Anil Potti, Joseph R Nevins, Charles M Perou

**Affiliations:** 1 Department of Pharmacology and Toxicology, University of Utah, 112 Skaggs Hall, Salt Lake City, UT 84112, USA; 2 Duke Institute for Genome Sciences & Policy, Duke University Medical Center, 2121 CIEMAS, Durham, NC 27701, USA; 3 Lineberger Comprehensive Cancer Center, University of North Carolina, 102 Mason Farm Road, Chapel Hill, NC 27599, USA; 4 Department of Genetics, University of North Carolina, 120 Mason Farm Road, Chapel Hill, NC 27599, USA; 5 The Pulmonary Center, Boston University School of Medicine, 715 Albany St, Boston, MA 02118, USA; 6 Department of Pathology & Laboratory Medicine, University of North Carolina, Chapel Hill, NC 27599, USA; 7 Division of Hematology/Oncology, Department of Medicine, University of North Carolina, Chapel Hill, NC 27599, USA; 8 Carolina Center for Genome Sciences, 5016 Genetic Medicine Building, University of North Carolina, Chapel Hill, NC 27599, USA

## Abstract

**Introduction:**

Perhaps the major challenge in developing more effective therapeutic strategies for the treatment of breast cancer patients is confronting the heterogeneity of the disease, recognizing that breast cancer is not one disease but multiple disorders with distinct underlying mechanisms. Gene-expression profiling studies have been used to dissect this complexity, and our previous studies identified a series of intrinsic subtypes of breast cancer that define distinct populations of patients with respect to survival. Additional work has also used signatures of oncogenic pathway deregulation to dissect breast cancer heterogeneity as well as to suggest therapeutic opportunities linked to pathway activation.

**Methods:**

We used genomic analyses to identify relations between breast cancer subtypes, pathway deregulation, and drug sensitivity. For these studies, we use three independent breast cancer gene-expression data sets to measure an individual tumor phenotype. Correlation between pathway status and subtype are examined and linked to predictions for response to conventional chemotherapies.

**Results:**

We reveal patterns of pathway activation characteristic of each molecular breast cancer subtype, including within the more aggressive subtypes in which novel therapeutic opportunities are critically needed. Whereas some oncogenic pathways have high correlations to breast cancer subtype (RAS, CTNNB1, p53, HER1), others have high variability of activity within a specific subtype (MYC, E2F3, SRC), reflecting biology independent of common clinical factors. Additionally, we combined these analyses with predictions of sensitivity to commonly used cytotoxic chemotherapies to provide additional opportunities for therapeutics specific to the intrinsic subtype that might be better aligned with the characteristics of the individual patient.

**Conclusions:**

Genomic analyses can be used to dissect the heterogeneity of breast cancer. We use an integrated analysis of breast cancer that combines independent methods of genomic analyses to highlight the complexity of signaling pathways underlying different breast cancer phenotypes and to identify optimal therapeutic opportunities.

## Introduction

The practice of oncology continually faces the challenge of matching the right therapeutic regimen with the right patient, balancing relative benefit with risk to achieve the most favorable outcome. This challenge is often daunting, with marginal success rates in many advanced disease contexts, likely reflecting the enormous complexity of the disease process coupled with an inability to guide properly the use of available therapeutics. The clinical and molecular characteristics of an individual tumor are the result of multiple mutations acquired over time and continued evolution of the responses to environment, all of this in the context of the inherited germline variations that affect tumor development. The complexity of carcinogenesis thus leads to immense natural heterogeneity in tumor phenotypes, disease outcomes, and response to therapies.

New technologies offer the potential of genome-wide biologic data that may serve as powerful adjuncts to currently available clinical and biochemical markers in dissecting cancer biology. Integrating clinicopathologic variables with genome-wide data may begin to characterize the complexity of disease, thus identifying discrete subsets of pathology that have not been recognized before the use of genomic data. The ability to find structure in the data, in the form of patterns of gene expression that provide snapshots of gene activity in a cell or tissue sample at a given instant of time, is transforming biology from an observational science into a data-intensive quantitative science. The dimension and complexity of such data provide opportunity to uncover patterns and trends that can distinguish subtle phenotypes in ways that traditional methods cannot.

One approach is the use of DNA microarray analysis to define subgroups of breast cancer patients based on unique profiles of gene expression that have distinct clinical outcomes [1,2]. A particular focus has been placed on the basal-like subtype, which defines a group of patients with poor outcome. The Basal-like subtype is negative for expression of common pathologic measurements including estrogen receptor, progesterone receptor, and HER2 receptor. This is also the predominant subtype observed in cancers of patients with BRCA1 mutations [1]. In addition, the luminal B subtype also represents a poor-prognosis group with few therapeutic options. This subtype correlates positively with clinical measurements such as ER expression, high proliferation index, and poor tumor grade [3]. Other work has made use of gene-expression profiles to develop genomic signatures of cell-signaling pathways that can then serve as guides for directing the use of targeted therapeutic agents [4-6]. Additionally, expression signatures have also been developed to predict the sensitivity to a variety of standard-of-care cytotoxic chemotherapeutic drugs [7,8].

Although in principle these two approaches could be used independently, we also recognize the opportunity for synergy, whereby a combination of the two methods for analysis of breast cancer has the potential to dissect further the heterogeneity of the disease. Here we describe an integrated analysis of breast cancers that brings these distinct forms of expression analysis together, both to highlight the complexity evident within the subtypes and, at the same time, to identify therapeutic opportunities specific to each subtype.

## Materials and methods

We made use of three publicly available breast tumor data sets profiled on Affymetrix U133a microarrays: Wang and colleagues [9] [GEO:GSE2034], Miller and colleagues [10] [GEO:GSE3494], and Pawitan and associates [GEO:GSE1456]. We then applied multiple different expression predictors that used different methods. These predictors were all derived by using other microarray data sets, and thus, the data sets used here represent true test sets. These data sets were individually normalized by using DWD (Distance Weighted Discrimination) and then used as described later. Predicted subtypes, pathways, and chemosensitivities were performed on each data set independently, and then these results were combined to form a single data set for analysis.

### Analysis of expression data to define intrinsic subtypes

Breast cancer subtypes were assigned as described in Hu and others [2]. The training set of 259 samples representing the five subtypes (Luminal A, Luminal B, HER2-enriched, Basal-like, and Normal-like) and 306 genes previously identified were used to build a corresponding set of five centroids [2]. The test sets were first adjusted for platform effects by centering each feature to have a median log2 value of zero within each set. Features were assigned Entrez Gene identifiers, and duplicate identifiers were collapsed to the mean. Each test case was then compared with the five standardized centroids by using Spearman's rank correlation. A test case was then assigned the subtype of the nearest centroid.

### Analysis of expression data for predicting pathway activation

Pathway analysis made use of previously described methods [4,6]. In brief, before statistical modeling, gene-expression data are filtered to exclude probe sets with signals present at background noise levels and for probe sets that do not vary significantly across samples. Data sets are normalized prior to binary regression using distance weighted discrimination [11]. A signature represents a group of genes that together exhibit a consistent pattern of expression in relation to an observable phenotype. Each signature summarizes its constituent genes as a single expression profile and is here derived as the first principal component of that set of genes (the factor corresponding to the largest singular value), as determined by a singular value decomposition. Given a training set of expression vectors representing two biologic states, a binary probit regression model is estimated by using bayesian methods. Importantly, when predicting the pathway activation of cancer cell lines or tumor samples, the gene selection, identification, and regression model are based on the training data only and then projected into the test data such as additional cell-line or tumor-expression data. This leads to evaluations of predictive probabilities of each of the two states for each case in the validation set. Bayesian fitting of binary probit regression models to the training data permits an assessment of the relevance of the gene-expression signatures in within-sample classification and provides an estimation and uncertainty. Relative predictions from the binary regression models are centered, and, in some cases, hierarchic cluster is performed by using Gene Cluster 3.0 [12].

In addition to the pathway signatures of Bild and associates [6], four additional pathways were studied; however, they were analyzed by using a different method. Specifically, we used the estrogen-regulated genes from Oh and colleagues [13], the 52-gene TP53 mutation signature from Troester and associates [14], the HER1 pathway 2 signature from Hoadley and colleagues [15], and the proliferation signature from Whitfield and others [16]. Each set of genes was split into those induced or repressed in the experimental state of the original publication, and only those features shown to be induced in the experimental state were considered here. The sample-specific score for each of these gene sets was assigned as described in Park and co-workers [17]. The features are standardized to have a mean of zero and unit variance. The mean of the features in each set was taken to be the sample specific score for that pathway.

### Chemosensitivity signatures

Because the expression signatures and the patient data were based on multiple platforms, all these signatures were consolidated to the Affymetrix HG-U133A platform by using *ChipComparer*. An initial training set representing two biologic states (drug resistant and drug sensitive) constitutes a chemosensitivity signature with binary regression methods performed in R/Bioconductor. Class labels (zero or one) were assigned to those that fall into two distinct clusters. Bayesian fitting of binary probit regression models to the training data then permits an assessment of the relevance of the metagene signatures in within-sample classification, and estimation and uncertainty assessments for the binary regression weights mapping metagenes to probabilities. To guard against overfitting, given the disproportionate number of variables to samples, leave-one-out cross-validation analysis was performed to test the stability and predictive capability of the model. Predictions of chemosensitivity (in the validation samples involving the Wang and Miller cohorts) were then evaluated by using methods previously described [7] producing estimated relative probabilities and associated measures of uncertainty across the validation samples. Hierarchic clustering of tumor predictions was performed by using R/Bioconductor. All the data analyses were implemented by R/Bioconductor statistical packages [18] and GraphPad Prism software (GraphPad Software Inc., La Jolla, CA, USA).

## Results

### Gene expression subtypes in breast cancer

**Figure 1:**
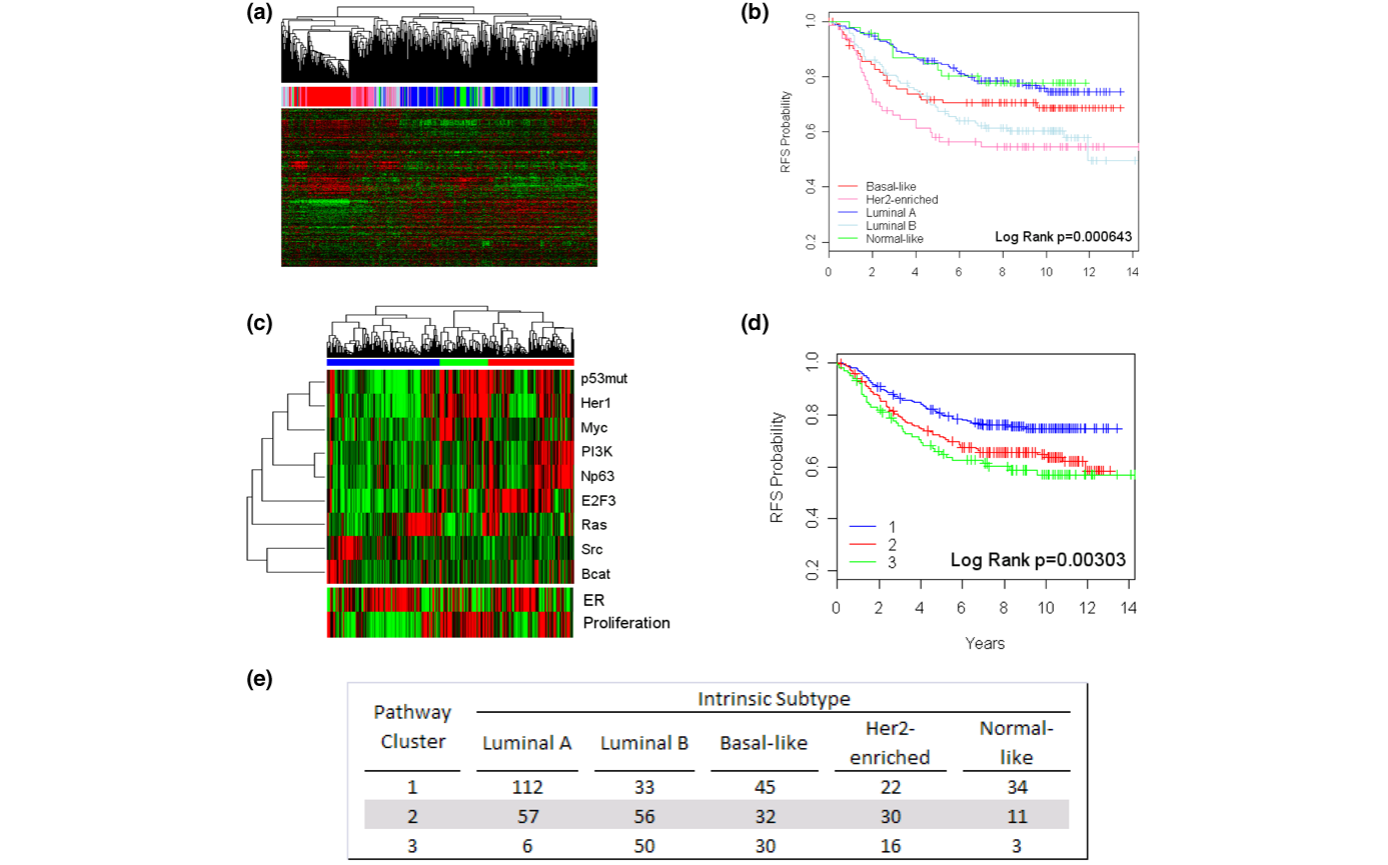
Dissection of breast cancer heterogeneity by using intrinsic subtypes and pathway patterns. **(a) **Hierarchic cluster analysis from a combined dataset comprising 537 samples. The centroid subtype predictions are shown immediately below the dendrogram with red for Basal-like, green for Normal-like, Luminal A as dark blue, Luminal B as light blue, and pink as HER2-enriched. **(b) **Kaplan-Meier survival plot for relapse-free survival by using the centroid predictions. **(c) **Cluster analysis of pathway-activation status predictions with red indicating active status, black, average pathway status, and green, low to absent pathway-activation status. **(d) **Kaplan-Meier survival plot based on the clustering of pathways from (c). **(e) **Correlations between tumor subtype (a) and groupings based on pathway status (c).

By using hierarchic clustering of gene-expression data, we previously showed that breast tumors can be classified into five major subtypes (Luminal A, Luminal B, HER2-enriched, Basal-like, Normal Breast-like). Importantly, further work has shown that these subtypes are reproducible across different sample sets and can predict relapse-free and overall patient survival times [1,2,19,20]. For this study, we made use of a combined data set, as described in Methods, that uses samples taken from Wang and co-workers [GEO:GSE2034] and Miller and associates [GEO:GSE3494]. Figure 1a shows a typical hierarchic clustering using the genes defining the intrinsic subtypes. Subtype predictions were generated as described in Hu and colleagues [2], and these predictions were in agreement with segregation in the sample dendrogram. As expected, the subtype predictions were associated with relapse-free survival times with the Luminal A and Normal-like groups indicative of good prognosis (Figure 1b).

The expression characteristics of these subtypes are consistent with previous observations including an HER2^+ ^expression cluster that is also predominantly ER^-^, and that contained multiple genes from the 17q11 amplicon, including HER2/ERBB2 and GRB7. A basal expression cluster also was present and contained genes (that is, c-KIT, FOXC1, and P-Cadherin) previously identified to be characteristic of basal epithelial cells. A Luminal/ER^+ ^expression cluster was present and contained ER, XBP1, FOXA1, and GATA3. GATA3 was recently shown to be somatically mutated in some ER^+ ^breast tumors [21].

### Patterns of pathway deregulation in breast cancer

The analysis of breast cancer expression data by unsupervised methods to reveal structure in the data set makes no assumptions regarding the underlying biology that might define the various subtypes. Our recent studies described a strategy making use of gene-expression signatures that reflect the activity of various oncogenic signaling pathways that can be used to characterize the status of important signaling pathways in tumors and to relate this to clinical outcome, as well as the potential for predicting response to targeted therapeutic agents [4,6,8]. We have now made use of these pathway signatures to aid in characterizing breast cancer subtypes, and predictions of pathway activation were performed by using each of the previously described pathway signatures [6]. Additional signatures developed independently for HER1 [15], ER [13], p53 [14] pathways, and a proliferation-signature [16], were used as described in the Methods section and also were tested. These results are displayed as a heat map reflecting the relative activity of each pathway, in which red represents high activity (Figure 1c), with samples clustered according to these probabilities (ER and proliferation were excluded from the cluster analysis, but are shown for reference purposes).

Three prominent clusters of samples/tumors were identified by this analysis. As one measure of the relevance of the patterns of pathway activation, we examined the clinical outcome of patients identified by these patterns of pathway activation. As shown in Figure 1d, these patterns of pathway activity segregate samples into clinically relevant groups, as assessed by relapse-free survival (*P *= 0.007). The survival curves of the different groups appear similar to the subtype grouping, and in fact, the segregation of samples by pathway activity is significantly associated with intrinsic subtype (Figure 1e). This result also suggests that subtype identity is attributable, at least in part, to activation of some of these pathways.

### Dissection of intrinsic subtypes using signatures of pathway deregulation

**Figure 2:**
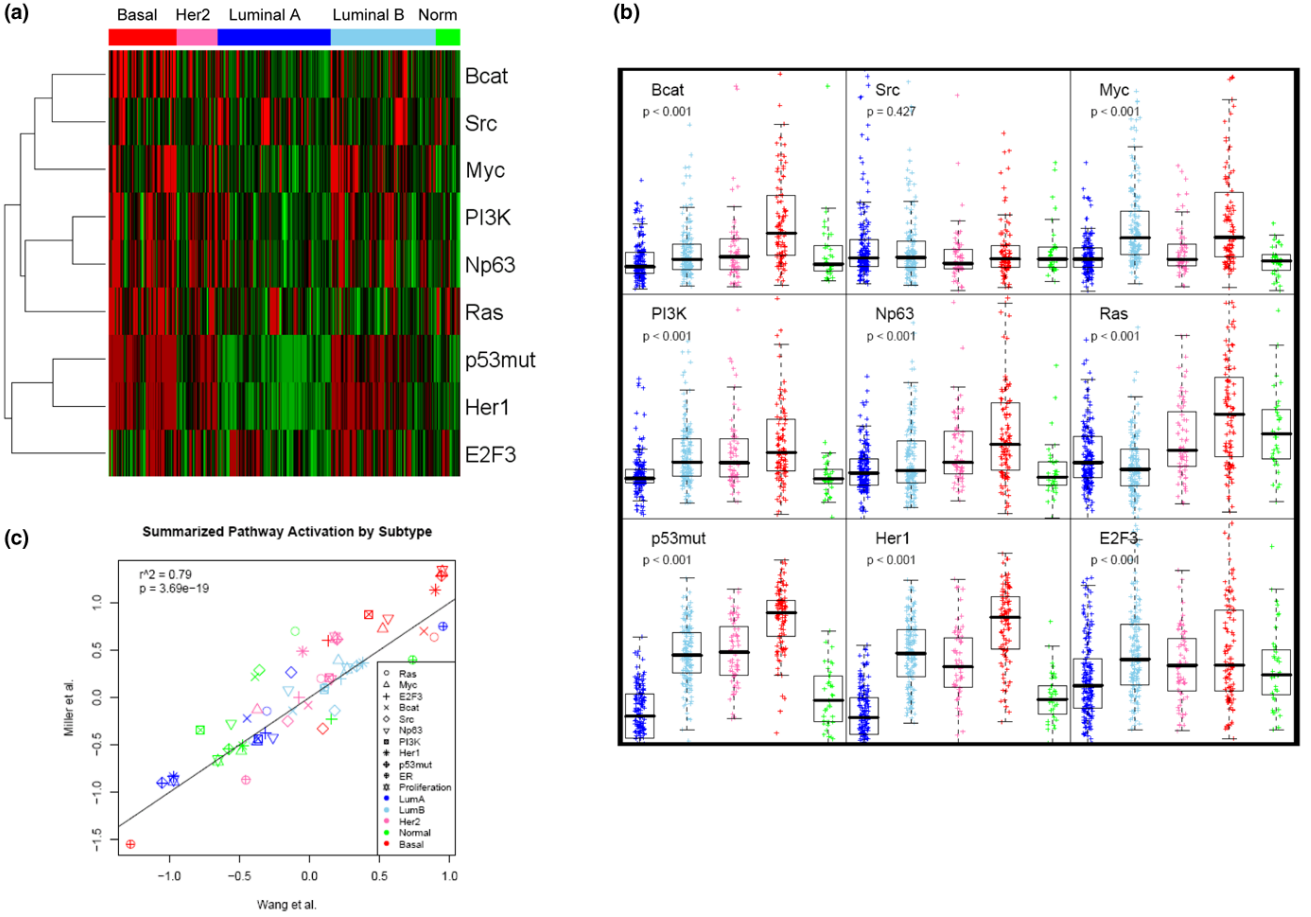
Pathway-activation status patterns are characteristic of intrinsic subtypes. **(a) **Heat maps of scaled pathway-activation scores in which the subjects are ordered according to their predicted subtype. The pathway-activation status of different pathways is displayed for the combined dataset, with strong pathway activation displayed by red, average status by black, and low to absent pathway activation by green. **(b) **Box-and-whisker plot showing pathway activation as a function of subtype displayed for the combined dataset. **(c) **Comparison of pathway activation as a function of subtype in the two datasets.

Although the division of breast cancers into the five subtypes defined by expression data is a clear first step in dissecting the heterogeneity of breast cancer, it also is likely that dissection within each subtype would further our understanding of breast tumor heterogeneity. Moreover, rather than using the gene-expression data or the pathway-signature data to define subtypes as parallel analyses, the real opportunity lies at the intersection of these two approaches, by using them in a complementary fashion. In the example shown in Figure 2a and quantified in Figure 2b, it is evident that distinct patterns of pathway activation can be seen as a function of subtype. For example, high predicted activity of RAS, CTNNB1 (β-catenin), TP53, and HER1 pathways was associated with the Basal-like subtype, in some cases consistent with previous observations [22-24]. Additionally, RAS pathway activation was seen in Basal-like, HER2-enriched, and Normal-like subytpes, whereas both Luminal A and Luminal B subtypes have low predicted RAS activity. Additionally, the highest E2F activity was seen in the Basal-like, HER2-enriched, and Luminal B subtypes, which is consistent with their higher proliferation rates compared with Luminal A and Normal-like tumors. Thus, our combining of distinct signatures onto a common test set has recapitulated known relations and identified new ones. Also, the Basal-like, HER2-enriched, and Luminal B subtypes are characterized by activation of multiple pathways in contrast to the Luminal A subtype, which show low predicted activity for most oncogenic pathways in this study.

These analyses were carried out with a set of samples that were combined from two separate data sets. Analysis of the results of pathway predictions in each data set separately demonstrated a close agreement in the patterns observed (Figure 2c), thus emphasizing the robustness of the measures. In addition, predictions of pathway activities in a third independent data set provided further confirmation for the patterns of pathway activity typical of a given intrinsic subtype [see Additional data file 1].

Each pathway also was examined for significance in univariate survival analysis across all patients, and within subtypes. The MYC, TP53, and proliferation signatures were significant predictors of outcomes across all patients and within the Luminal B and HER2-enriched subtypes. The ER and HER1 pathway signatures were prognostic across all patients, but ER was significant only within the Luminal B subtype, and HER1 trended toward significance in Luminal B (Table 1) with some showing significance for both (Table 1).

**Table 1:** Univariate association of pathway deregulation with disease-free survival

Pathway	Overall	LumA	LumB	Her2	Basal
Ras	0.64	0.081	0.6	0.75	0.87
Myc	*0.0033*	0.92	*0.0091*	*0.037*	0.7
E2F3	*1.8E-04*	0.55	*0.0044*	0.13	0.13
Bcat	0.22	0.43	0.084	0.64	0.56
Src	0.85	0.79	0.29	0.32	0.7
NP63	0.93	0.21	0.83	0.73	0.19
PI3K	0.72	0.12	0.82	0.84	0.15
p53mut	*6.0E-06*	0.15	*0.0014*	*0.037*	0.77
Her1	*1.5E-04*	0.12	0.077	0.11	0.98
ER	*3.9E-04*	0.15	*0.001*	0.083	0.078
Proliferation	*3.0E-05*	0.088	*0.0035*	*0.046*	0.76

*P *values of the pathway coefficient when predicting disease-free survival **(Cox proportional hazard *P *values)**. The pathways with significant relapse-free survival associations are indicated in italics; these were tested across all patients, and across all patients within each subtype separately.

Although specific pathways exist with an overall elevation in each subtype, it is clear that pathway status is not simply a function of intrinsic subtype. As is evident from Figure 2, heterogeneity exists when one examines each pathway pattern within a subtype. Heterogeneity of pathway status is the most evident within the Basal-like tumors, which demonstrate relatively large variation in the activation of many pathways. The Luminal B samples also encompass a broad range of activity across multiple pathways, but to a lesser extent than the Basal-like samples. Luminal A and Normal-like samples give the lowest variation in activity across the pathways.

**Figure 3:**
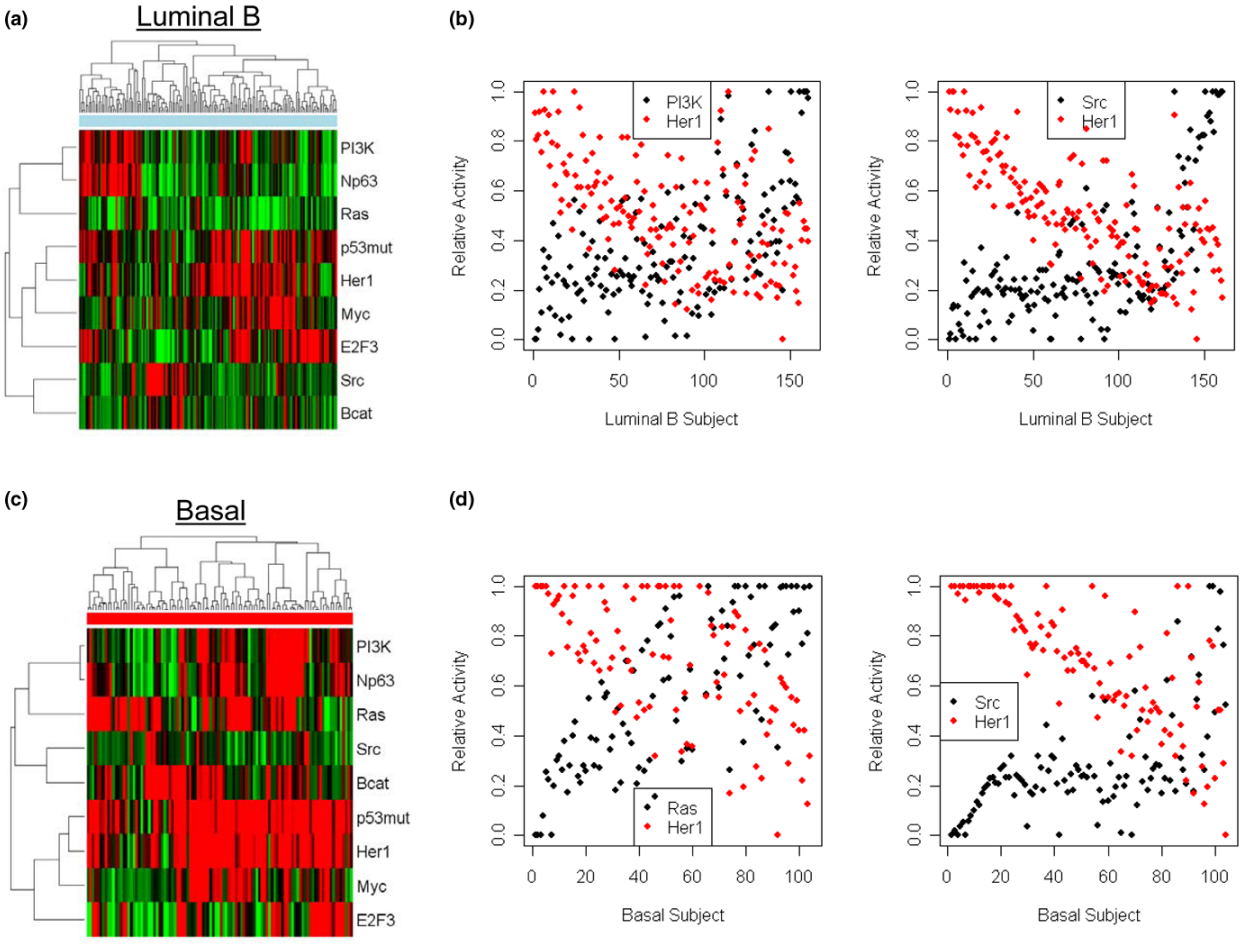
Inverse relations in pathway activation in Basal-like and Luminal B subtypes. Pathway-activation patterns are shown for **(a, b) **Basal-like, and **(c, d) **Luminal B subtypes. Relative pathway-activation status is shown on the y-axis, whereas each location on the x-axis represents an individual tumor.

Previous work showed that the prediction of pathway activation can coincide with sensitivity to drugs that target a component of the pathway [8,25,26]. As such, this information provides an opportunity to identify new therapeutic options for these patients by providing a potential basis for guiding the use of pathway-specific drugs. Therefore, we next focused on this heterogeneity as a mechanism to identify potential therapeutic opportunities that might be unique for individual subtypes. In particular, we examined the patterns of pathway activation in both the Luminal B and Basal-like subtypes, which represent patients with a poor prognosis and the need for new therapeutic approaches. In particular, we examined whether specific pathways had inverse or compensatory patterns within a subtype that could provide alternative therapeutic opportunities for patients. We found multiple instances in which patients exhibited inverse relations of activation of one pathway versus another. These results include complementary patterns for HER1 and PI3K, as well as for HER1 and SRC in the Luminal B data subtype (Figure 3a and 3b). Likewise, the HER1 and SRC pathways, or the HER1 and RAS pathways, show complementary patterns in the Basal-like subtype, in which patients exhibit activity of either pathway independent of the other (Figure 3C and 3D). Taken together, these analyses demonstrate that each of the intrinsic breast cancer subtypes associates with specific patterns of pathway activation. The pathways with highest correlation to the intrinsic breast cancer subtypes include TP53 and HER1. Additionally, oncogenic pathways can have high variability of activity within a specific subtype, such as MYC, E2F3 and SRC, reflecting biology independent of common clinical factors.

### Genomic signatures that predict response to cytotoxic chemotherapeutics

A further opportunity for identifying novel therapeutic opportunities makes use of a collection of expression signatures developed to predict sensitivity to commonly used cytotoxic chemotherapeutic agents. By using dose-response information and matched expression data on the NCI-60 cell lines, we developed a panel of gene-expression signatures representing resistant and sensitive patterns for a series of cytotoxic agents commonly used in the treatment of solid tumors, including breast cancer [7]. Further work has shown that several of these predictors can accurately predict the response to the drugs in patients [7,8,27].

**Figure 4:**
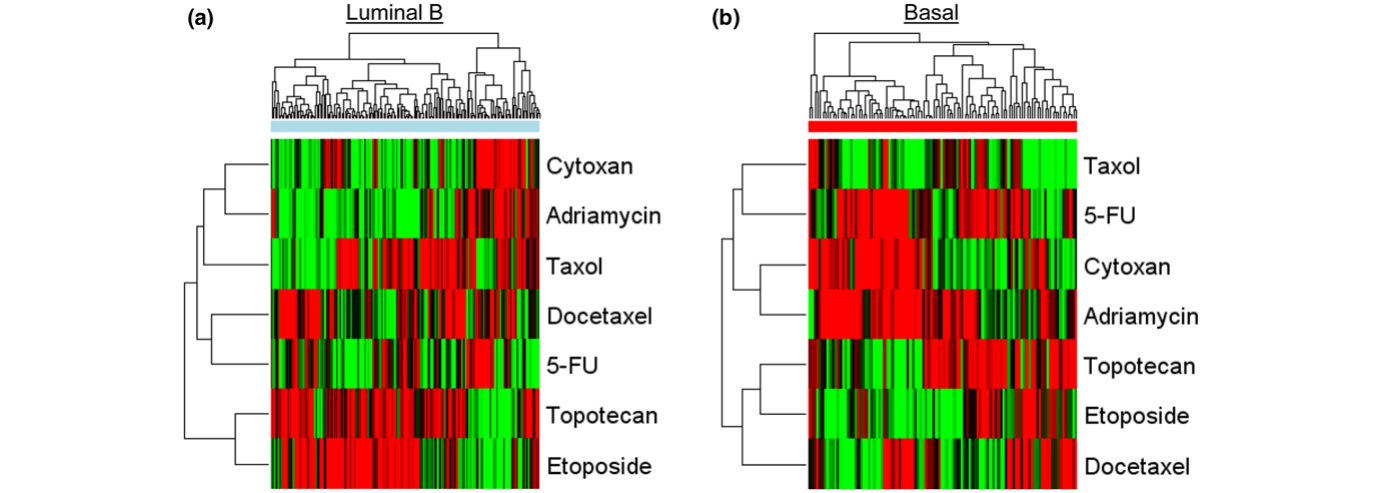
Chemotherapeutic sensitivity profiles for Luminal B and Basal-like subtypes. Heat maps of scaled chemotherapeutic-sensitivity predictions in the combined dataset for **(a) **Luminal B and **(b) **Basal–like subtypes are shown. Samples were clustered, based on the predicted sensitivities.

We made use of these chemotherapy-response signatures to predict the likely sensitivity to these agents within these two breast cancer sample sets. Again, we focused on the Basal-like and Luminal B subgroups, given the need for new and novel therapeutic options for these patients. As shown in Figure 4, both the Basal-like and Luminal B subtypes exhibited predicted sensitivity to many of the commonly used cytotoxic agents. Perhaps most important, clear evidence of heterogeneity was found within each of these subgroups with respect to predicted sensitivity to these drugs. These data provide a further indication of the heterogeneity of tumors within the subtypes, underscoring the need to focus on this heterogeneity and to identify therapeutic options tailored to the individual patient. In addition, the analysis also suggests opportunities for matching drugs with patients with similar inverse relations as seen with the pathway signatures. For example, in the Basal-like subtype, patients that are predicted to be sensitive to doxorubicin (Adriamycin) are predicted to be resistant to topotecan, and *vice versa*. An additional opportunity afforded by the availability of signatures predicting sensitivity for multiple cytotoxic agents, as well as pathway activation, is the identification of potential combinatorial strategies that would derive from overlaps in the predicted sensitivities.

## Discussion

Since the advent of chemotherapy to treat cancer, numerous advances have been made in the development, selection, and application of these agents; sometimes with remarkable successes, as seen in the case of combination chemotherapy for lymphomas or platinum-based therapy for testicular cancers. Present-day therapeutic regimens derive from prospective clinical studies that evaluate the relative effectiveness of regimens for groups of patients. Importantly, these studies by necessity have used largely unselected populations of patients and thus represent effective regimens for the group, but not necessarily for any one individual patient. In short, it is clear that breast cancer is not a single disease but rather a collection of diseases with unique characteristics. Individualizing treatments by identifying patients who will or will not respond to specific agents will potentially increase the overall effectiveness of these drugs and limit the incidence and severity of toxicities that impair the functional status of patients and their ability to tolerate further therapies.

Toward this goal, we have used gene-expression profiles derived from DNA microarray analysis to dissect the heterogeneity evident within human breast cancers. This includes the ability to identify subtypes of cancer that can be associated with distinct clinical characteristics and outcomes, and the ability to assay for the activity of specific signaling pathways. The value in these studies is to identify disease subtypes that represent more homogeneous collections of tumors and patients, so as the better to approach opportunities for individualized therapeutics. The results we present here extend our initial observations several important steps by combining complementary methods to characterize further the intrinsic subtypes with respect to pathway activation and potential chemotherapy sensitivities.

With the goal of identifying therapeutic opportunities that match the characteristics of individual breast cancer patients, we primarily focused on the Basal-like and Luminal B subtypes, given the poor prognosis of these groups of patients. Moreover, given the fact that the Basal-like subtype is characterized as HER2-not amplified, PR^- ^and ER^-^, existing therapeutic opportunities are limited. Our analysis from using both pathway profiles and chemotherapy-sensitivity profiles suggests multiple possible strategies for therapeutic opportunities in Basal-like and Luminal B patients. A large fraction of the Basal-like subgroup exhibits HER1 pathway activation, which we have observed before by using other data sets [15], and which could be matched with a variety of EGFR inhibitors; in fact, clinical trials using HER1 inhibitors on metastatic "triple-negative/basal-like" patients are under way (ClinicalTrials.gov Identifier: NCT00232505 and NCT00248287); these findings lend strong support to our approach and suggest the potential for a general strategy of using patterns of pathway activation as a means of identifying therapeutic opportunities. Interestingly, it is also clear that an inverse pattern of pathway deregulation exists for HER1 and the SRC pathways, as well as for HER1 and RAS pathways within the Basal-like subtype, suggesting an opportunity to direct therapy, either HER1 specific, SRC specific, or RAS specific, as a function of these pathway profiles. The Basal-like subtype is also enriched for tumors predicted to be sensitive to doxorubicin, and a complementary pattern was found with predicted sensitivity to topotecan, suggesting additional opportunities for combination therapies that might be effective in this subtype.

The Luminal B subtype also was characterized by activation of the HER1 pathway along with complementary activation of the PI3K pathway or SRC pathway. Many agents in development target PI3K or SRC, and further options exist for activities downstream of the action of PI3K, such as AKT1 and mTOR. A fraction of the Luminal B tumors were also predicted to be sensitive to doxorubicin, although less so than for the Basal-like subtype. Nevertheless, a complementary sensitivity exists with etoposide, suggesting an opportunity to make use of these two agents.

## Conclusions

**Figure 5:**
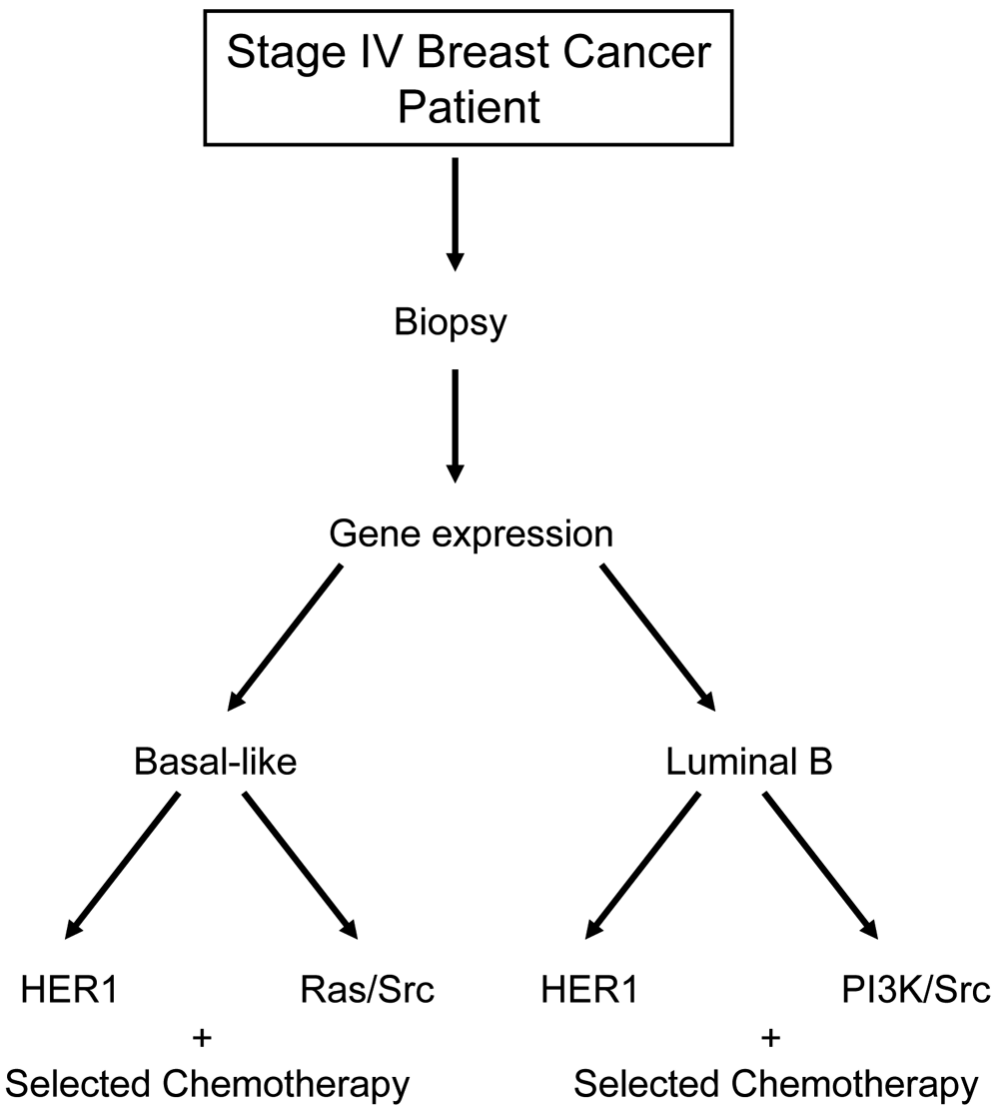
Schematic diagram of a potential genomics-guided trial based on intrinsic subtype and pathway signatures.

We believe that our studies could guide the development of prospective clinical studies that would evaluate the efficacy of therapeutic regimens, based on a combined pattern of pathway and chemosensitivity within a given subtype (Figure 5). The initial stratification would be on the intrinsic subtype classification, with further dissection based on pathway or chemosensitivity signatures or both, with the latter two providing guidance in the choice of therapeutics. Given the poor prognosis and the lack of current therapeutic options, we believe that a focus on the Basal-like and Luminal B subtypes is of highest priority.

## Abbreviations

ER: estrogen receptor; PR: progesterone receptor; RMA: Robust Multichip Average.

## Competing interests

CP has an equity interest in University Genomics. The PAM50 intrinsic subtyping assay evaluated in this article is the focus of a patent application from UNC, and CP and JP are listed as inventors. All other authors have no competing interests.

## Authors' contributions

AB and AG were responsible for the oncogenic-pathway predictions. JP and KAH were responsible for the ER, HER1, p53, and proliferation-pathway predictions and data analysis. AP was responsible for chemotherapy-response signatures. JN, AB, JP, AP, LS, CA, LC, and CP were responsible for project planning and data analysis. JN, JP, CP, and AB were responsible for writing the manuscript.

## Supplementary Material

Additional file 1 A box-and-whisker plot showing pathway activation as a function of subtype displayed for an additional validation dataset. Click here for file
