# Supplementary figures and images for: An integration of complementary strategies for gene-expression analysis to reveal novel therapeutic opportunities for breast cancer
Source: Breast Cancer Res. 2009 Jul 28;11(4):R55. doi: 10.1186/bcr2344 (PMC2750116; doi:10.1186/bcr2344)

Supplemental Figure 1

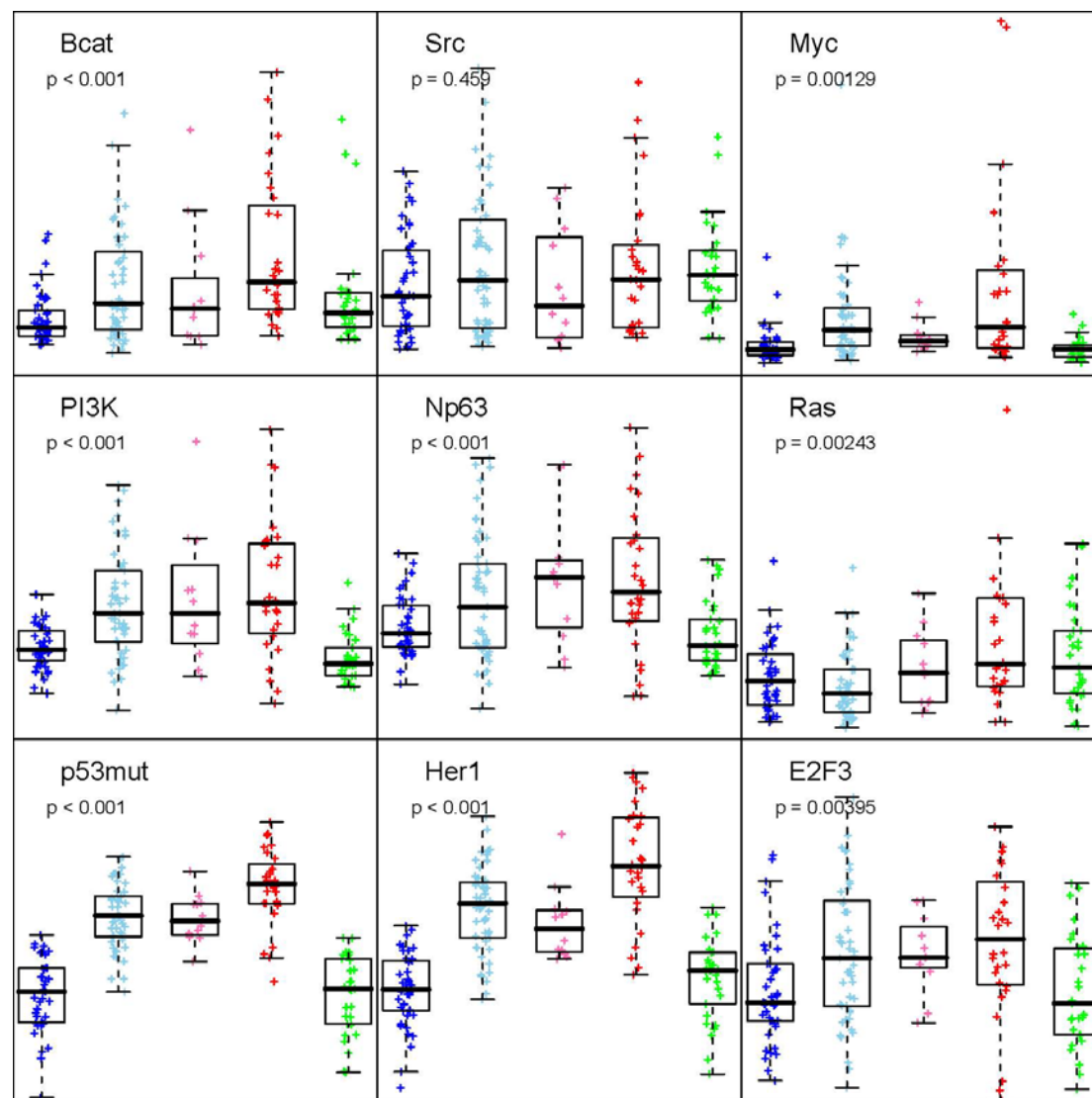

Supplement: Additional file 1 — A box-and-whisker plot showing pathway activation as a function of subtype displayed for an additional validation dataset. [file bcr2344-S1.pdf]
